# Sexing Live Pupae and Adults of Two Wax Blooming Beetles, *Colposcelis microderoides microderoides* and *Anatolica polita borealis*

**DOI:** 10.1673/031.013.13601

**Published:** 2013-11-27

**Authors:** Yan Wang, Fuchun Zhang, Ji Ma

**Affiliations:** Xinjiang Key Laboratory of Biological Resources and Genetic Engineering, College of Life Science and Technology, Xinjiang University, 14 Shengli Road, Urumqi, 830046, China.

**Keywords:** cleft, 8th sternite, papilla, sexual dimorphism, sexing method

## Abstract

The wax blooming beetles *Colposcelis microderoides microderoides* Reitter and *Anatolica polita borealis* Kaszab (Coleoptera: Tenebrionidae) are small, flightless beetles living in the Guerbantonggut desert in the northwestern region of China. These beetles were recently found to have wax blooms, and their short life cycle and ease of rearing under laboratory conditions make them excellent models for advanced studies on desert adaptation. To date, dissection has been used for sex identification in these species, whereas a nondestructive method is needed for further studies on sexual dimorphism. Microscopic examinations of pupae and adults revealed distinct differences between the sexes in the 8th abdominal sternites, including the presence of a setose cleft in adult males that is not visible in females, in which the 8th sternite forms a single semicircular plate. The female pupae have a pair of tan papillae and a tan genital orifice, which are absent on the male 8th sternite. These two sexual characteristics can be used to sex live adults and pupae. These methods are simple, nondestructive, 100% accurate, and useful for sex differentiation of dead beetles and some other tenebrionid species (including some pests) in both the field and the laboratory.

## Introduction

The wax blooming beetles *Colposcelis microderoides microderoides* Reitter and *Anatolica polita borealis* Kaszab (Coleoptera: Tenebrionidae) are small, flightless beetles in the Tentyriini tribe. They live in the Gurbantonggut desert, the second largest desert in the northwestern region of China (Huang et al. 2005). *C. m. microderoides* and *A. p. borealis* adopt several strategies to survive in hostile, arid environments (Chen et al. 2007; Qiu et al. 2010). The study of desert beetles is important because it illustrates many of the solutions evolved by arthropods to the problems engendered, in an extreme form, by life in terrestrial environments (Cloudsley-Thompson 2001). We have successfully reared these beetles under laboratory conditions. Moreover, their abundance, short life cycle, and ease of rearing under laboratory conditions make them excellent models for advanced ecology, molecular biology, and physiology studies of desert adaptations of darkling beetles.

**Figure 1.**
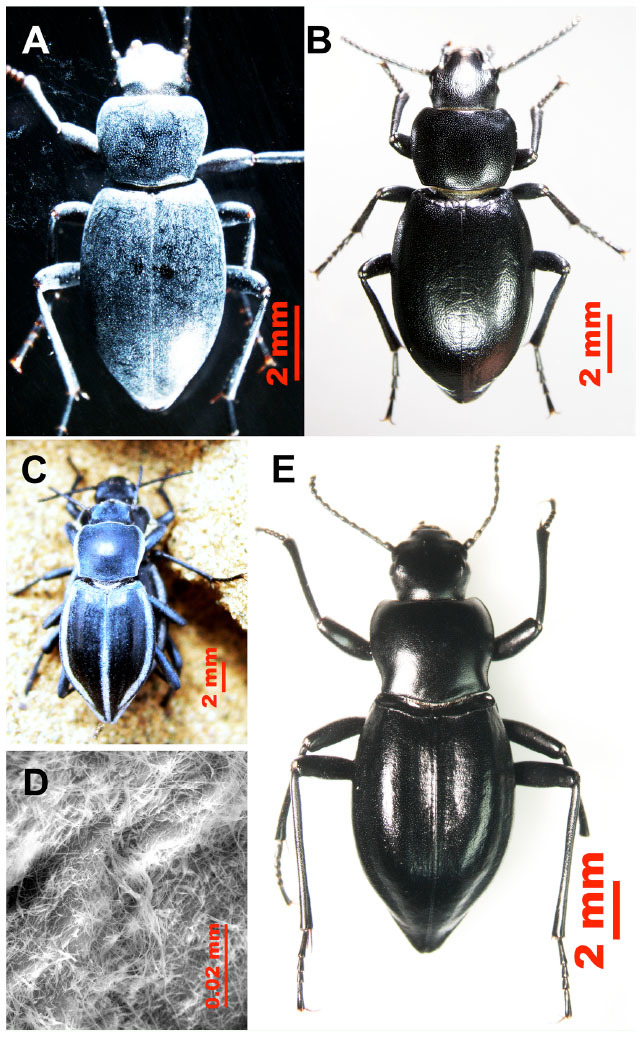
Dorsal view of the color phase and wax secretion of the desert beetles *Colposcelis microderoides microderoides* (A and B) and *Anatolica polita borealis* (C, D, and E). (A) Light blue color phase (low humidity); (B) black color phase (high humidity); (C) light blue color phase (in the field); (D) high magnification view of individual cuticle surfaces showing the wax layer created by wax filaments; (E) black color phase (in the laboratory). High quality figures are available online.

Recently, it was found that these beetles exhibit the wax blooming phenomenon (Figure 1) in which reversible colors are brought about by wax blooms. Several desert adaptations have been demonstrated in response to wax blooms, including protection from predators, microorganisms, ultraviolet light, and mechanical abrasion by the substrate (McClain et al. 1985).This phenomenon is not normally found in arthropods. A few tenebrionid beetles in the Namib and Sonoran deserts are reported to have reversible pastel wax blooms (McClain et al. 1991). Those beetles belong to the Adesmiini, Zophosini (McClain et al. 1985), and Cryptoglossini (Hadley 1979) tribes. They have an extracuticular wax bloom covering either part of or their entire body surface. In contrast to the beetles from the Adesmiini, Zophosini, and CryptoglossiniRecently, it was found that these beetles exhibit the wax blooming phenomenon (Figure 1) in which reversible colors are brought about by wax blooms. Several desert adaptations have been demonstrated in response to wax blooms, including protection from predators, microorganisms, ultraviolet light, and mechanical abrasion by the substrate (McClain et al. 1985).This phenomenon is not normally found in arthropods. A few tenebrionid beetles in the Namib and Sonoran deserts are reported to have reversible pastel wax blooms (McClain et al. 1991). Those beetles belong to the Adesmiini, Zophosini (McClain et al. 1985), and Cryptoglossini (Hadley 1979) tribes. They have an extracuticular wax bloom covering either part of or their entire body surface. In contrast to the beetles from the Adesmiini, Zophosini, and Cryptoglossini tribes, *C. m. microderoides* and *A. p. borealis* live in a different geographic range (the Guerbantonggut desert in Asia instead of the Namib and Sonoran deserts) and they belong to the tribe Tentyriini.

Riddle et al. (1976) showed that desert arthropods displayed both osmotic regulation and tolerance patterns that varied by sex. *Alphitobius diaperinus* females exhibited significantly longer survival times than males under desiccating conditions (Renault and Coray 2004), and the supercooling temperature points were significantly different between adult males and females (Salin et al. 2000). Further study of tenebrionid beetles has the potential to add to the understanding of sexual dimorphism, which is an important consideration in both basic and applied research (Posada et al. 2011). In order to conduct further studies on sex-related differences, one needs to know the sexes of live individuals. Unfortunately, identification of the sex of tenebrionid beetles has typically been determined through dissection (Salin et al. 2000). Thus, the development of nondestructive methods for sex identification is needed to facilitate further studies of sexual dimorphism in *C. m. microderoides* and *A. p. borealis* as well as in tenebrionid beetles as a whole.

Attempts to sex adults by squeezing the abdomen (Pszczolkowski et al. 2008) or withdraw the genitalia from the abdomen with forceps (Vinod et al. 2008) have usually resulted in the damage to or death of the beetles (Bhattacharya et al. 1970). The lack of discreet, gender-specific externally visible characteristics, such as hairs and spines (Innocenzi et al. 2002), a sex patch (Hinton 1942; Faustini et al. 1981), or a visible intersegmental membrane (Bhattacharya et al. 1970), makes sex determination of the adults difficult, which is common among tenebrionids (Vinod et al. 2008).

Morphological characteristics have been used for sexing Coleopteran pupae (Halstead 1963; Bhattacharya et al. 1970; Sugiyama et al. 1996; Wang et al. 2006). However, the features reported, although similar, are not exactly the same in all the species examined, or even in all the species of the same group (Wang et al. 2006).

Little is known about the sexual dimorphism of either pupae or adult *C. m. microderoides* and *A. p. borealis*. Here we report the morphological details that are sex-specific in both pupae and adults of these species, and therefore their sex can be determined nondestructively, permitting sexing of the living pupae and adults with 100% accuracy without injuring or killing the insects.

## Materials and Methods

### Insects

*C. m. microderoides* and *A. p. borealis* adults were originally hand picked in 2008 from Wujiaqu (44° 29′ N, 87° 31′E, 410 m a.s.l.), which is about 100 km northeast of the geological center of Asia. The insects were maintained at 30 ± 0.5 °C, 30 ± 6% RH, and a 16:8 L:D photoperiod. The adult rearing, egg collection, larval rearing, and pupal collection were conducted as previously described (Wang et al. 2011). The pupae and adults used in these experiments were obtained from both field collections and from colonies maintained under laboratory conditions.

### Examining the parameters with potential for use in identifying the sex nondestructively

Pupae and adults were examined under an SMZ-800 stereomicroscope (Nikon, http://www.nikoninstruments.com) and a Quanta 250 FEG Scanning Electron Microscope (FEI, http://www.fei.com) to identify any differences in body size, body contours, color pattern, elytra, the head, compound eyes, the antennae, the legs, the thorax, and the abdominal plates (Duan et al. 1999). The characteristic that seemed to have the greatest potential was the nature of the 8th abdominal sternites of both the pupae and adults.

To observe the 8th abdominal sternites of the adults, each beetle was placed on the stage of a stereomicroscope (15 × magnification) equipped with Elements 3.0 software (Nikon SMZ-800, www.nikon.com) with the ventral part of the beetle facing upward (the head was positioned away from the observer). Then a polished, narrow needle such as that in a 5 ml syringe that was filed to make it blunt (Shanghai Zhiyu Medical Material Co., Ltd., http://zhiyumedical.globalimporter.net) was used. The polished narrow tip of the needle was inserted between the elytra and the last visible abdominal sternite, about 1.5 mm from the tip of the abdomen. By sliding the tool slightly beyond the apex of the abdomen (i.e., on the posterior side closest to the observer) and gently lifting, the edges of the 7th sternite and 7th tergite were separated (Sappington and Spurgeon 2000), which exposed the posterior region of the 8th sternite.

The pupae were divided into two groups according to the morphological differences of the 8th abdominal sternites. The two groups of pupae were kept under laboratory conditions (30 ± 0.5 °C, 30 ± 6% RH, and a 16:8 L:D photoperiod) and were allowed to develop to the teneral adult stage. A confirmation of the sex was carried out by an inspection of the adult genitalia with the posterior side closest to the observer. In the field, the observations were conducted with the assistance of an eye loupe (15 ×).

## Results and Discussion

After examining hundreds of *C. m. microderoides* and *A. p. borealis* adults and dozens of pupae, no distinctive differences between males and females in body size, contours, color, elytra, heads, compound eyes, antennae, legs, or thorax plates could be found. Duan et al. (1999), Sappington and Spurgeon (2000), and Innocenzi et al. (2002) were also unable to find distinguishing characteristics. Therefore, these structures may not reliably be used for sex differentiation in these species.

**Figure 2.**
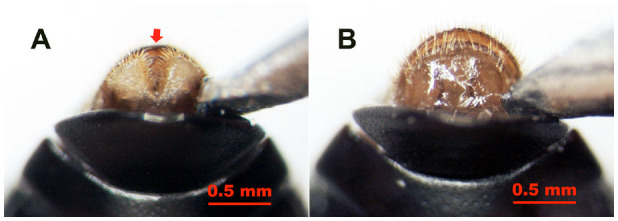
Ventral view of *Colposcelis microderoides microderoides* adults being sexed by probing (pressing the outer abdominal segments with a blunt, polished, narrow syringe needle), showing the posterior region of the 8th sternite with the setose median cleft (red arrow) in a male (A) and without the cleft in a female (B). The color of the posterior margin of the 8th sternite in males (A) is lighter than that in females (B). High quality figures are available online.

**Figure 3.**
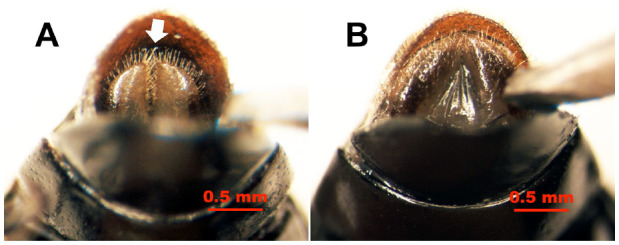
Ventral view of *Anatolica polita* borealis adults being sexed by probing (pressing the outer abdominal segments with a blunt, polished, narrow syringe needle),showing the posterior region of the 8th sternite with the setose median cleft (white arrow) in a male (A) and without the cleft in a female (B). The posterior margin color of the male 8th sternite (A) is lighter than that of females (B). High quality figures are available online.

**Figure 4.**
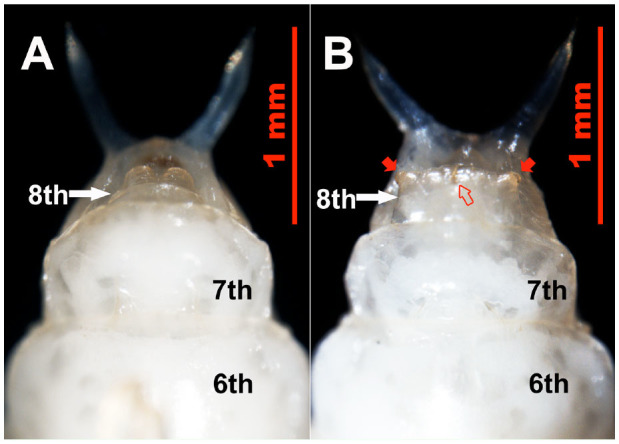
Ventral view of *Colposcelis microderoides microderoides* pupae, showing the different shapes of the 8th sternite (white arrows) between males (A) and females (B). Abdominal segment numbers are shown. In male pupae, the 8th sternite is small and narrow, with two bumps caudal to it, while the 8th sternite of female pupae is large and flat, with a pair of papillae (red arrows) and a genital orifice (red outlined arrow). High quality figures are available online.

**Figure 5.**
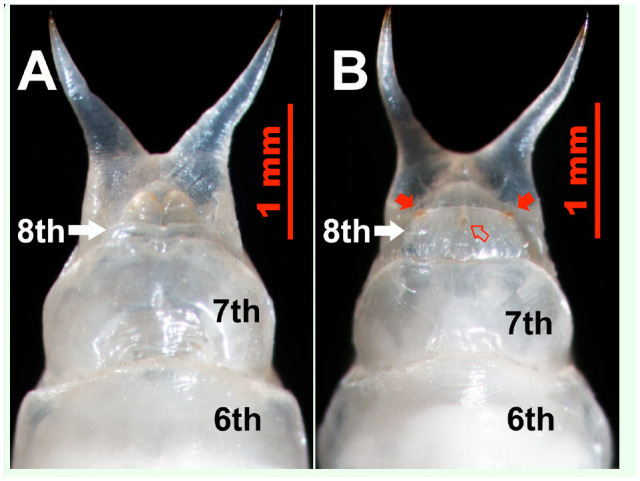
Ventral view of *Anatolica polita borealis* pupae, showing the different shapes of the 8th sternites (white arrows) between males (A) and females (B). The abdominal segment numbers are shown. In the male pupae, the 8th sternite is small and narrow, with two bumps caudal to it, while the 8th sternite of the female pupae is large and flat, with a pair of papillae (red arrows) and a genital orifice (red outlined arrow). High quality figures are available online.

However, it was found that male and female pupae and adults could be conclusively distinguished based on differences of the ventral parts of the 8th abdominal sternites (Figures 2, 3, 4, and 5). In adults, the 8th abdominal sternites of the males are clearly split by a setose median cleft (Figures 2A and 3A), but the median cleft is not seen in females because the 8th abdominal sternite is a single, large semi-circlular plate (Figures 2B and 3B). In addition, the colors of the posterior regions of the 8th sternites in males (Figures 2A and 3A) are white and lighter than those of females (Figures 2B and 3B).

These characteristics could be easily seen by using the probing method described above (Figures 2 and 3). It is necessary to fully expose the diagnostic clefts on the 8th sternites but unnecessary to see the whole cleft and its setae. The setaceous cleft was clearly observed on the posterior margin of the male 8th sternite, so when the posterior edge of the 8th sternite could be seen, the probing was sufficient. We refer to this sexing method as the sternal cleft probing method.

Male and female *C. m. microderoides* and *A. p. borealis* adults could be completely distinguished by sternal cleft probing without exception (n = 600). During the observations, it was not necessary to squeeze the abdomens of the insects (Pszczolkowski et al. 2008) or withdraw the abdominal sternite from the abdomen with forceps (Vinod et al. 2008). Therefore, this method reduced the likelihood of damaging the insects and had the advantage of keeping the insects alive after sexing for successive experiments.

In the male pupae, the 8th abdominal sternites were small and narrow (Figures 4A and 5A), and each had two large carnose bumps caudal to it, while in female pupae, the 8th sternites were large, wide, and flat (Figures 4B and 5B), with each having a pair of tan, semitransparent papillae and a tan genital orifice (Figures 4B and 5B). Based on these sexual characteristics, a method that we termed the sternal papilla method was used for nondestructive sexing of live pupae. This method permitted sex differentiation with complete accuracy (n = 80).

**Figure 6.**
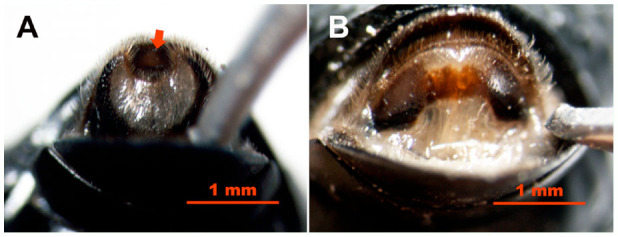
Ventral view of *Adesmia anomala dejeani* adults being sexed by probing (pressing the outer abdominal segments with a blunt, polished, narrow needle), showing the posterior region of the 8th sternite with the setose median cleft (red arrow) in a male (A) and without the cleft in a female (B). The cleft of the male 8th sternite is semicircular and surrounded by a round white area, which is absent in females (B). High quality figures are available online.

The methods described above for both pupae and adults will be useful for sex identification of live wax blooming beetles in both the laboratory and the field. These methods can also be used to easily and accurately differentiate dead males and females of adult and pupal *C. m. microderoides* and *A. p. borealis*. Additionally, we have found that these same methods can be applied to adults and pupae (live and dead) of some other species of desert beetles such as *Adesmia anomala dejeani* Gebler (Coleoptera: Tenebrionidae), in both the laboratory and field conditions (n = 60) (Figures 6 [adults] and 7 [pupae]).

**Figure 7.**
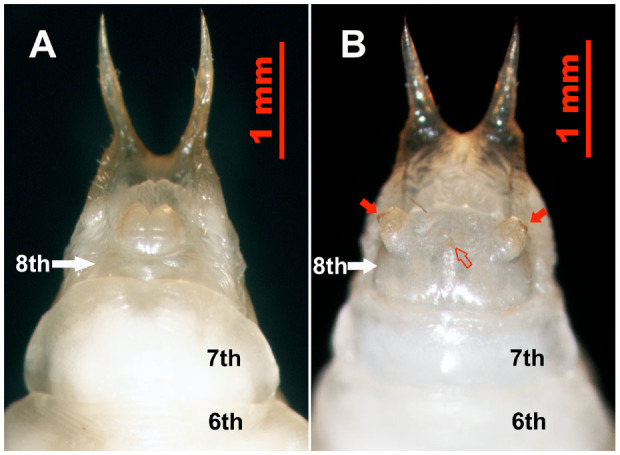
Ventral view of *Adesmia anomala dejeani* pupae, showing the different shapes of the 8th sternite (white arrows) in males (A) and females (B). The abdominal segment numbers are shown. In male pupae, the 8th sternite is small and narrow, with two bumps caudal to it, while the 8th sternite of female pupae is large and flat, with a pair of papillae (red arrows) and a genital orifice (red outlined arrow). High quality figures are available online.

It appears, however, that the characteristics used to distinguish the sexes in the Tenebrionidae family vary depending on the tribe the species comes from. For example, the clefts of beetles from the Tentyriini tribe (*C. m. microderoides* and *A. p. borealis*) look like slits, but the clefts of beetles from the Adesmiini tribe (*Adesmia anomala dejeani*) look like notches (Figure 6A), similarly to *Luprops tristis* (Vinod et al. 2008). Differences among tribes are also apparent in the pupal papillae, which is relatively larger in beetles from the Adesmiini tribe than in those from the Tentyriini tribe (Figure 7B). Additionally, within the Tentyriini tribe, the color patterns of the 8th sternite can vary. For instance, unlike *C. m. microderoides* and *A. p. borealis*, *Microdero punctipennis* male and female adults were similar in color, while the 8th sternites of male *Sternoplax soltvorowiana* adults were darker than those of females.

Due to the small size of some beetles (< 8 mm), a binocular microscope or an eye loupe (15 × magnification) is generally required to accurately analyze the 8th abdominal sternites. However, some large beetles (> 15 mm), such as *S. soltvorowiana* and *A. a. dejeani*, could be sexed with the naked eye.

These two methods based on the differences in the 8th abdominal sternites may be by far the simplest and least destructive methods that can be used to accurately differentiate between the sexes of both adult and pupal tenebrionid beetles, such as *C. m. microderoides*, *A. p. borealis*, *A. a. dejeani*, *Tenebrio molitor*, etc. Hopefully these methods will be widely used by others in need of nondestructive techniques for sex determination of both pupae and adult tenebrionid beetles (including pests in the fields of agriculture, forestry, and stored products).
